# Supplementary material for: Speech cortical activation and connectivity in typically developing children and those with listening difficulties
Source: Neuroimage Clin. 2022 Aug 28;36:103172. doi: 10.1016/j.nicl.2022.103172 (PMC9467868; doi:10.1016/j.nicl.2022.103172)
Supplement: Supplementary Data 1 [file mmc1.pdf]

Speech cortical activation and connectivity in typically developing children and those with listening difficulties

Abbreviated title: Cortical speech networks and listening

Hannah J. Stewart [1, 2, 3], Erin K. Cash [2], Lisa L. Hunter [2], Thomas Maloney [4], Jennifer Vannest [2,5] and David R. Moore [2, 6, 7]

1. Division of Psychology and Language Sciences, University College London, London, UK
2. Communication Sciences Research Center, Cincinnati Children's Hospital Medical Center, Cincinnati, Ohio, USA
3. Department of Psychology, Lancaster University, Lancaster, UK
4. Pediatric Neuroimaging Research Consortium, Cincinnati Children's Hospital Medical Center, Cincinnati, Ohio, USA
5. Department of Communication Sciences and Disorders, University of Cincinnati, Ohio, USA
6. Department of Otolaryngology, College of Medicine, University of Cincinnati, Cincinnati, Ohio, USA
7. Manchester Centre for Audiology and Deafness, University of Manchester, Manchester, M13 9PL, UK

Correspondence: Hannah J. Stewart
Department of Psychology
Lancaster University
Lancaster UK
h.stewart5@lancaster.ac.uk

Keywords: listening difficulties, pediatric, fMRI, resting state, connectivity, speech perception

Table 1: Developmental disorders in each group, as reported by the children's caregivers. (ASD: autism spectrum disorder; ADHD: attention deficit hyperactivity disorder; SLP: speech language pathologist).

Group	N	ASD	ADHD	Dyslexia	Seen a SLP
LiD	43	4	21	1	11
TD	41	0	0	0	0

Table 2: Brodmann area (BA) and MNI coordinates for maximum intensity voxels from the fMRI speech listening task contrasts. Cortical activation is across all 85 participants as groups were not significantly different after correcting for multiple comparisons. All three contrasts showed large areas of activation covering the auditory cortex. With activation extending out towards language (Wernicke's), memory (parahippocampal) and decision making (frontal orbital cortex) areas as the contrasts progressed from simpler stages of speech listening (phonology) to more advanced (semantics). Cluster threshold = 4.0 voxels, p-FDR = .95. See Figure 2.

**Contrast**	**Max. intensity MNI coordinates**				**Brain Regions (Harvard-Oxford atlas)**	**Cluster size (voxels)**
	**BA**	**x**	**y**	**z**		
**Phonology** Rotated > Rotated+Vocoded	21	-48	0	-24	L middle temporal gyrus	1603
	21	60	0	-16	R middle temporal gyrus, ant	1238
	41	-40	-34	6	L planum temporale	7
**Intelligibility** Clear > Rotated	20	-46	12	-34	L temporal pole	2567
	21	50	8	-30	R temporal pole	1114
	48	-50	18	18	L inferior frontal gyrus	6
**Semantics** Clear > Rotated+Vocoded	20	-44	6	-34	L frontal pole	4351
	20	46	8	-32	R temporal pole	2810
	20	-36	-10	-46	L temporal fusiform cortex, post	33
	38	-52	26	-10	L frontal orbital cortex	25
	37	-38	-40	-22	L temporal fusiform cortex, post	7

Table 3: Brodmann area (BA) and MNI coordinates for the regions of interest from the fMRI task used in the rs-fMRI ROI-to-ROI analysis. Cluster threshold = 4.0 voxels, p-FDR = .95.

Contrast	ROI	Max. intensity MNI coordinates BA	x	y	z	Brain Regions (Harvard-Oxford atlas)	Cluster size (voxels)
**Phonology** Rotated > Rotated+Vocoded	1	21	66	-18	-10	R middle temporal gyrus	317
	2	22	-60	-42	6	L supramarginal gyrus, post	23
	3	21	66	-4	-6	R superior temporal gyrus	351
	4	21	-60	-16	-10	L middle temporal gyrus, post	192
	5	22	60	-28	10	R planum temporale	5
	6	21	52	-4	-10	R superior temporal gyrus, ant	17
	7	21	-48	-2	-14	L planum polare	319
	8	21	52	4	-14	R superior temporal gyrus, ant	60
	9	21	-54	0	-10	L superior temporal gyrus, ant	153
	10	21	-48	4	-22	L temporal pole	25

	11	41	-40	-34	6	L planum temporale	19
	12	21	-60	-28	-6	L middle temporal gyrus, post	330
	13	21	60	0	-16	R middle temporal gyrus, ant	356
	14	21	-68	-28	2	L superior temporal gyrus, post	400
	15	21	-48	0	-24	L middle temporal gyrus, ant	149
	16	21	60	-22	-6	R middle temporal gyrus, post	131
**Intelligibility** Clear > Rotated	1	21	60	-20	-6	R middle temporal gyrus, post	11
	2	21	-60	-46	2	L middle temporal gyrus, temporoccipital part	131
	3	21	50	8	-30	R temporal pole	210
	4	20	-46	12	-34	L temporal pole	82
	5	21	66	-4	-6	R superior temporal gyrus, ant	37
	6	20	-52	-18	-14	L middle temporal gyrus, post	208
	7	20	44	6	-26	R temporal pole	25
	8	21	-46	-4	-22	L superior temporal gyrus, ant	255
	9	38	58	12	-18	R temporal pole	64

	10	21	-52	0	-10	L superior temporal gyrus, ant	264
	11	20	-42	12	-28	L temporal pole	223
	12	21	-44	-40	6	L supramarginal gryus, post	9
	13	21	-44	-48	-2	L inferior temporal gyrus	28
	14	21	-50	-34	-6	L middle temporal gyrus, post	635
	15	21	52	0	-26	R middle temporal gyrus, ant	576
	16	21	-68	-28	2	L superior temporal gyrus, post	116
	17	20	-48	-4	-26	L middle temporal gyrus, ant	573
	18	38	-50	24	-12	L frontal orbital cortex	24
	19	20	50	-20	-16	R middle temporal gyrus, post	180
	20	48	-50	18	18	L inferior frontal gyrus, pars opercularis (Broca's)	6
**Semantics** Clear > Rotated+Vocoded	1	21	68	-20	-10	R MTG, post	301
	2	21	-52	-50	2	L middle temporal gyrus, temporoccipital part	363
	3	20	46	8	-32	R temporal pole	344
	4	20	-44	6	-34	L temporal pole	185
	5	21	66	-4	-6	R superior temporal gyrus, ant.	420

6	20	-52	-18	-16	L middle temporal gyrus, post	309
7	20	44	0	-26	R STG, ant	108
8	20	-46	-8	-22	L MTG, post	593
9	21	48	8	-18	R temporal pole	210
10	21	-52	0	-10	L superior temporal gyrus, ant	368
11	20	-42	8	-30	L temporal pole	314
12	21	-42	-32	2	L planum temporale	86
13	21	-50	-48	2	L middle temporal gyrus, temporoccipital part	128
14	21	-50	-34	-8	L MTG, post	899
15	21	54	-6	-26	R middle temporal gyrus, ant	792
16	21	-68	-28	2	L superior temporal gyrus, post	419
17	20	-48	-6	-26	L middle temporal gyrus, ant	634
18	38	-52	26	-10	L inferior frontal gyrus, pars traingularis	24
19	20	-48	-2	-30	L middle temporal gyrus, ant	12

	20	20	52	-18	-16	R middle temporal gyrus, post	625
	21	20	52	-6	-26	R middle temporal gyrus, ant	6
	22	20	-44	-6	-26	L inferior frontal gyrus, ant	10
	23	20	-36	-10	-46	L temporal fusiform cortex	33
	24	37	-38	-40	-22	L temporal fusiform cortex, post	4

Table 4: Mild to moderate correlations were found between temporal connectivity and behavioural outcome measures (auditory processing skills, verbal vocabulary, selective attention, switching attention and episodic memory). Note that only connections with significant group differences (Figure 4) are shown here.

**Network**	**ROI # – ROI #**	**Behavioral outcome**	**r (77)**	**p**
Intelligibility	14-14	SCAN auditory figure ground	.33	.004
		NIH verbal vocab	.25	.028
		NIH task switching	.26	.024
Speech	24-7	SCAN complete words	.30	.011
		SCAN complete sentences	.30	.010
		NIH verbal vocab	.31	.006
